# “Factors associated with non-small cell lung cancer treatment costs in a Brazilian public hospital”

**DOI:** 10.1186/s12913-018-2933-0

**Published:** 2018-02-17

**Authors:** Carla de Barros Reis, Renata Erthal Knust, Claudia Cristina de Aguiar Pereira, Margareth Crisóstomo Portela

**Affiliations:** 1 Escola Nacional de Saúde Pública Sergio Arouca, Fundação Oswaldo Cruz, Rua Leopoldo Bulhões 1480, 7o andar, Rio de Janeiro, RJ 21041-210 Brazil; 2 Instituto Nacional de Câncer José Alencar Gomes da Silva – INCA, Praça da Cruz Vermelha 23 – 4o andar, sala 64. Centro. Rio de Janeiro, Rio de Janeiro, RJ 20230-130 Brazil

**Keywords:** Lung cancer, Hospital costs, Non-small cell lung cancer (NSCLC), Public hospital

## Abstract

**Background:**

The present study estimated the cost of advanced non-small cell lung cancer care for a cohort of 251 patients enrolled in a Brazilian public hospital and identified factors associated with the cost of treating the disease, considering sociodemographic, clinical and behavioral characteristics of patients, service utilization patterns and survival time.

**Methods:**

Estimates were obtained from the survey of direct medical cost per patient from the hospital’s perspective. Data was collected from medical records and available hospital information systems. The ordinary least squares (OLS) method with logarithmic transformation of the dependent variable for the analysis of cost predictors was used to take into account the positive skewness of the costs distribution.

**Results:**

The average cost of NSCLC was US$ 5647 for patients, with 71% of costs being associated to outpatient care. The main components of cost were daily hospital bed stay (22.6%), radiotherapy (15.5%) and chemotherapy (38.5%). The OLS model reported that, with 5% significance level, patients with higher levels of education, with better physical performance and less advanced disease have higher treatment costs. After controlling for the patient’s survival time, only education and service utilization patterns were statistically significant. Individuals who were hospitalized or made use of radiotherapy or chemotherapy had higher costs. The use of these outpatient and hospital services explained most of the treatment cost variation, with a significant increase of the adjusted R^2^ of 0.111 to 0.449 after incorporation of these variables in the model. The explanatory power of the complete model reached 62%.

**Conclusions:**

Inequities in disease treatment costs were observed, pointing to the need for strategies that reduce lower socioeconomic status and population’s hurdles to accessing cancer care services.

## Background

Cancer is a major public health problem worldwide and some 27 million incident cases and 12.6 million deaths from the disease are estimated for the year 2030 [1]. Increased incidence of the disease has been attributed to behaviors and lifestyles associated with economic development and urbanization, such as tobacco use, poor diet and physical inactivity, and an increased number lung, breast, colon and rectum cancers are being observed, especially in emerging economies [2, 3]. Regarding lung cancer, estimates indicate growth in the number of new cases between 2009 and 2020, namely, 40.3% in low-income countries and 38.4% in lower middle-income countries. In upper middle-income and high-income countries, growth level is expected at 23.4% and 24.2%, respectively [3].

Following the global trend, Brazil has experienced significant changes in the profile of diseases that affect the population, with a progressive increase of incidence and mortality from chronic degenerative diseases. According to the Ministry of Health, cancer was the second leading cause of death in 2013 after cardiovascular diseases [4]. About 420,300 new cases of cancer are estimated for 2016, excluding non-melanoma skin cancer, of which 28,200 are trachea, bronchus and lung cancer cases [5].

Lung cancer is the most common of all malignant tumors, with a yearly increase of 2% in its worldwide incidence. It has become one of the leading causes of preventable death, since it is very much associated with smoking. Diagnosis usually occurs in advanced stages, since symptoms in the early stages of the disease are not common, increasing the likelihood of debilitating symptoms, failure of interventions and unfavorable outcomes [6]. The disease remains highly lethal, with mortality/incidence ratio of approximately 0.90 [5]. Non-small cell lung cancer (NSCLC) accounts for around 85% of all lung cancer cases [7, 8].

Besides human losses caused by cancer-related deaths, patient care financial and economic costs are high, not only due to disease burden faced by the health system, but also the incorporation of new increasingly expensive technologies. The challenge becomes even greater in inadequate funding scenarios, unequal distribution of resources and services and increased need for investments [9, 10]. Addressing suffering and disease care financing hardships depends on planning control actions [9] and can benefit from a greater understanding of varying disease treatment costs.

The present study aims to identify factors associated with direct cost variation of care for advanced non-small cell lung cancer, considering demographic, socioeconomic, clinical and behavioral characteristics of patients and the pattern of the service utilization at a reference hospital of the Unified Health System (SUS) in Rio de Janeiro, Brazil.

## Methods

### Study subjects

The study was based on secondary data from a retrospective cohort of patients diagnosed with NSCLC stage IIIB / IV enrolled in the Chest Department, José Alencar Gomes da Silva National Cancer Institute (INCA), from January 1 to December 31, 2011. INCA is a public hospital in the city of Rio de Janeiro, Brazil inserted into the structure of the universal, comprehensive and equitable Brazilian public health system, namely, the Unified Health System (SUS). In addition to being linked to the Ministry of Health as a High Complexity center of reference providing direct and free care to cancer patients, the hospital is responsible for the implementation of the National Ontological Care Policy at the national level.

### Sample

We have considered 251 patients over 18 years of age with NSCLC with no second primary tumor, diagnosed in the advanced stages of the disease (IIIB/IV), with performance status from 0 to 2, who were not clinical trial participants and were diagnosed and treated at INCA. Specifically, it is worth clarifying that the patient’s performance rating scale is used to assess disease progress and its impact on the patient’s daily life abilities, ranging from 0 (fully active patient) to 5 (death) [11].We have analyzed the total cost of treatment throughout an observation period of 18 months from the date of patient’s hospital screening. Of all patients, 85% died within the study observation period. Thus, costs underestimation for the remaining 15% is likely. The number of patients corresponds to 90% of an initial cohort of 277 subjects, which also included 26 patients whose monitoring was interrupted before the first year of observation [12]. This follow-up loss may have been motivated by different factors, such as the abandonment of treatment or transfer of care to the private health services network, for example.

### Costs estimates

Estimates were performed from the per patient cost survey, from the perspective of INCA - Cancer Hospital I - as a service provider of reference of the Unified Health System (SUS). Only direct costs have been included in the analysis, namely, drugs, consultations, administration of infusion protocols, services and procedures rendered, laboratory, imaging tests, blood transfusion and hospitalization. We did not consider information relating to non-medical direct costs or costs relevant to other perspectives of the analysis, such as disbursements of patients or family members (out-of-pocket costs). Transportation and food costs were not taken into account, nor were indirect costs, such as lost productivity due to inability to work resulting from treatment or loss of economic productivity related to premature death.

Data were collected from medical records and information systems available that enable inventory control, input, output and consumption of all materials and services offered at the hospital, in addition to registration and scheduling of patients. Some supplies are consumed in fixed proportions treated as kits. Kits’ components were established through consultation with professional experts responsible for related procedures, as well as with standard operating procedures and regulatory instructions of services provided by the hospital. Cost estimation implies the identification and measurement of resources used and their valuation thereof. This valuation resulted from the average unit cost or in accordance with a unified price list of procedures, strategic medicines and strategic supplies of the SUS. Thus, costs are expressed by multiplying quantities consumed and average unit value [13]. In the case of hospitalization, in addition to drug consumption and procedures performed, we also considered the hospital daily bed stay cost, information obtained from the Brazilian Federation of Hospitals (FBH). This amount included components such as bed and bath linen, patient’s hygiene and oral diet and nursing care. Amounts were converted into US dollars at year 2011exchange rate(R$ 1 = US$ 0.597).

### Statistical analysis

**Fig. 1:**
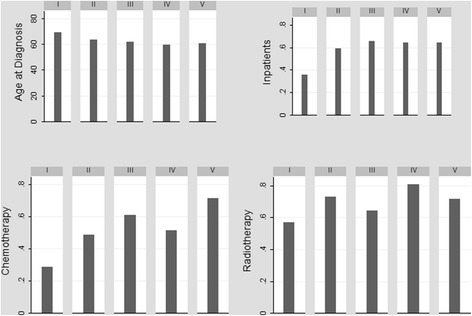
NSCLC Treatment Cost and Cost Logarithm Distributions– INCA 2011. Source: Authors’ own work. Amounts in 2011 US Dollars. Cost statistics: Average = US$ 5647. Median = US$ 3796. Mode = US$ 1371. Kurtosis = 21.30.Asymmetry = 4.14. lnCost statistics: Average = 5.11. Median = 5.23. Mode = 4.62. Kurtosis = 4.08. Asymmetry = − 0.47

The analysis of cost determinants was based on linear regression models estimated by the ordinary least squares (OLS) method, with gradual insertion of explanatory variables groups. In order to consider the skewed distribution of the “NSCLC treatment cost” dependent variable, we proceeded to its logarithmic transformation. This transformation seems to have been effective in reducing data’s high asymmetry, as shown in the Kernel density histograms and their central tendency (Fig. 1).

Six nested models are analyzed to verify the linear relationship between the treatment cost logarithm and potential explanatory variables, as follows: Model 1 – including only demographic characteristics of patients; Model 2 - Model 1 and socioeconomic variables; Model 3 – Model 2 and lifestyle variables; Model 4 –Model 3 and clinical variables; Model 5 –Model 4 and variables related to the use of outpatient and hospital services; and Model 6 –Model 5 and death period of the patient. Among demographic characteristics of patients are gender, age at diagnosis, race / color and marital status. Education is the only socioeconomic status-related variable. Behavioral variables considered are alcohol consumption and tobacco use. Disease stage (IIIB or IV), existing comorbidities and physical performance – measured in terms of performance status (0 to 2) – reflect the clinical conditions of patients. Hospitalization and chemotherapy / radiotherapy dummy variables assign value equal to one for patients who use these services and zero for those that do not. The analysis also considers the effect of patients’ survival time. Controlling this variable in the analysis is interesting since it helps to ascertain the extent to which other explanatory variables have independent effects on cost variation, which is influenced by the time the patient remains alive and, thus, receives treatment. Part of the costs incurred during the treatment of patients with better clinical conditions or that make use of chemotherapy / radiation therapy, for example, may result from a longer patient monitoring time by the hospital.

The estimated coefficients are interpreted as the percentage change in the average cost of treatment for each unit change in the independent variable. In the case where the regression factor is a dummy variable, this interpretation is given from the antilogarithm of the estimated coefficient subtracted from the unit [14].

With regard to inferences, we consider a significance level of α = 0.05. We analyze the statistical significance of the explanatory variables based on Student t-test. Model adjustments and comparisons are made through the adjusted R^2^ determination coefficient, which considers in its conformation models’ varying levels of freedom. White’s heteroskedasticity test [14, 15] and VIF statistics were performed to verify the homoscedasticity hypothesis of residuals and the multicollinearity of covariates, which are important for model efficiency and performance of statistical inference. *Non-rejection of the null constant variance hypothesis allows both generalized linear model and OLS estimation to be efficient, yielding reliable standard errors and t-tests.* Managing multiple databases with the various cost components was performed with the Statistical Analysis System (SAS®) version 9.3b, while analyses presented here were conducted using Stata 15.0 (StataCorp, College Station, TX).

## Results

### Patients’ characteristics

About 63% of patients were aged between 50 and 69 years on entering the service. Minimum and maximum age recorded was 36 and 86, respectively, with an average of 63 years. A prevalence of male patients (63%) was noted. Regarding race / color, 65% of patients were reportedly white, 30% mixed race and 5% black. Approximately half of the patients studied (49.8%) had not completed elementary school, 26.7% completed elementary school, 12.4% completed high school, and 5.5% completed higher education. Illiterate patients represented 5.5% of the sample. As for the considered lifestyle habits, about 90% and 49% of patients had a history of tobacco use and reported alcohol consumption throughout life, respectively. Approximately 50% of patients had some type of comorbidity; 33.5% were diagnosed at stage IIIB – locally advanced – and 66.5% at stage IV – metastatic disease. Most patients (57.0%) of the study had limited physical condition at the onset of the observation period, but were still able to perform light work (PS = 1). Almost 30% were able to take care of themselves, but were unable to carry out any work duties (PS = 2). Only 13.2% of patients were considered totally active and were able to maintain full and unrestricted pre-disease performance (PS = 0). From the sample of 251 patients, 27.5% died in less than three months, 50.6% in less than 6 months and 85% in less than 18 months.

### Treatment costs

**Fig. 2:**
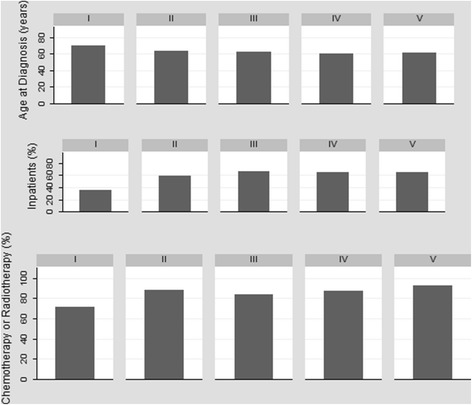
Percentage distribution of the components of the total NSCLC treatment cost – INCA 2011. Source: Authors’ own work

The cost of treatment per patient ranged between US$ 61 and US$ 54,278, with an average of US$ 5647 and a median of US$ 3796. Figure 2 shows that main components of the total cost were chemotherapy (38.5%), hospital daily bed stay (22.6%) and radiotherapy (15.5%), followed by drugs (10. 8%), imaging and laboratory tests (8.4%), procedures (2.3%), consultations (1.9%) and blood transfusion (0.6%) (Fig. 2). Approximately 70% of the total cost is related to outpatient care. A quasi-monotonic relationship between the patient’s time period to death and outpatient and hospital average costs has been observed. Table 1 shows that the greater the survival time of the patient, the greater the average outpatient costs associated with treatment of the disease are. Patients who survive for a longer period of time are more exposed to curative or palliative interventions. Notwithstanding this relationship is not so strong for hospitalization costs, the reverse correspondence is observed, possibly indicating that patients who die quicker are those most debilitated and in a more advanced stage of the disease, as they are hospitalized very shortly after diagnosis. The group of patients who died after the observation period (18 months) has the highest average and median outpatient costs, the lowest average and median hospitalization costs and greater variability of its values against the average, given by the coefficient of variation (Table 1).

**Table 1:** Outpatient and hospital costs according to death period of the patient – INCA, 2011

Death period	n	Outpatient Costs				Hospitalization Costs			
(months)		Median	Average	s.d.	c.v.	Median	Average	s.d.	c.v.
< 3	69	611	711	454	0.64	1666	2051	1949	0.95
3 to 6	58	1247	1594	1295	0.81	715	1,62	1939	1.20
6 to 9	32	2313	2919	2323	0.80	1606	1876	2105	1.12
9 to 12	26	3293	3943	3275	0.83	1666	1844	2077	1.13
12 to 18	28	3,33	4231	3637	0.86	833	1396	1742	1.25
> 18	38	5691	14,647	17,479	1.19	0	592	1364	2.30

Source: Authors’ own work

Amounts in 2011 US Dollars

Note: Standard Deviation (s.d.) and Coefficient of Variation (c.v.)

**Fig. 3:**
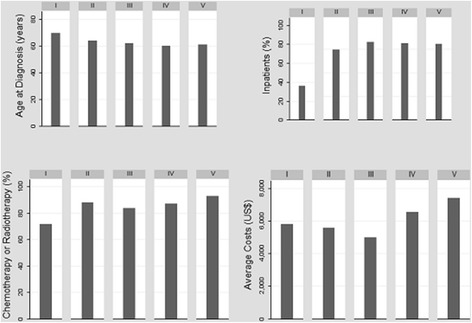
Age at diagnosis, proportion of health services use and average costs by level of education – INCA, 2011. Source: Authors’ own work. Note: I – Illiterate; II – Elementary Incomplete; III – Elementary; IV – High School; V – Higher Education

The correlation matrix of the predictors presents the pairwise correlation coefficients and the chi-square test of statistical significance in Table 2. As can be observed, besides the fact that the variables have weak correlation among themselves, few coefficients are statistically different from zero. Male patients have a higher correlation with alcohol and tobacco use throughout life and are older at diagnosis, compared to women. Patients with lower levels of education or who present some comorbidity or who are not hospitalized also present higher ages at the time of diagnosis. The highest correlation coefficients are observed for the relationships between the patient’s death period and their clinical conditions and use of health services. The longer the survival time, the better the patient’s physical condition, the greater the use of chemotherapy or radiotherapy services and smaller the likelihood of hospitalization (Table 2, Fig. 3).

**Table 2:** Correlation Matrix of the Covariates

	Age at Diagnosis	Man	Education	Alcohol	Tobacco	Comorbidity	PS	Staging	Hospitalization	Chemot/Radio	Death period
Age at Diagnosis	1.00										
Man	0.15**	1.00									
Education	−0.18***	0.03	1.00								
Alcohol	−0.08	0.13**	0.12	1.00							
Tobacco	−0.05	0.19***	0.09	0.17***	1.00						
Comorbidity	0.22***	−0.01	−0.06	0.09	−0.07	1.00					
PS	0.08	0.03	−0.06	0.03	0.05	−0.01	1.00				
Staging	−0.09	−0.07	− 0.06	− 0.02	− 0.10	0.08	0.02	1.00			
Hospitalization	−0.16**	− 0.06	0.09	− 0.05	0.05	−0.04	0.03	0.07	1.00		
Chemot/Radio	−0.03	0.05	0.05	0.02	0.01	0.09	−0.17***	−0.12	0.08	1.00	
Death period	0.01	0.06	−0.01	0.04	−0.05	0.08	−0.31***	− 0.19***	−0.29***	0.29***	1.00

Source: Authors’ own work

**p-value < 0.05 ***p-value < 0.01

Figure 3 shows the mean age at diagnosis, the proportion of hospitalized patients, the proportion of patients using chemotherapy or radiotherapy, and the average cost of treatment, by level of education. The descriptive analysis stratified by education allows us to observe that individuals with lower socioeconomic status have a diagnosis at more advanced ages and use less outpatient and inpatient services. Patients with high school or higher education have higher average costs of treatment than those with less schooling. These results may be expressing the greatest barriers to access to health services faced by the population of lower socioeconomic status, arriving at the time of diagnosis under more precarious health conditions, with greater severity of the disease and less chance of survival or to be treated. Although the results indicate the presence of inequities in the NSCLC treatment cost, a ceteris paribus analysis is necessary to isolate the effect of socioeconomic status from the other factors.

### Analysis of factors associated with treatment cost

Linear regression results are shown in Table 3. In model 1, in addition to the constant, only age at diagnosis is statistically significant and has a negative sign, indicating that patients with older age at diagnosis of the disease have lower treatment costs compared to other patients. Those with higher education levels have higher costs. Education and age variables remain significant for determining the treatment cost up to model 4. In models 5 and 6, only the group of patients with higher education has coefficient statistically different than zero. Both lifestyle-related variables, namely, tobacco use and alcohol consumption history are not statistically relevant for most models (Table 3).

**Table 3:** Multivariate linear regression models for the total NSCLC treatment cost logarithm – INCA, 2011 (*n* = 251)

	Model 1	Model 2	Model 3	Model 4	Model 5	Model 6
Age at diagnosis	−0.013 (0.005) ***	− 0.011 (0.005) **	− 0.011 (0.005) **	−0.010 (0.005) **	− 0.007 (0.004)	−0.004 (0.003)
Man	0.069 (0.095)	0.044 (0.096)	0.058 (0.100)	0.059 (0.097)	0.068 (0.077)	0.039 (0.064)
Color/race (reference: White)						
Black	−0.027 (0.202)	−0.025 (0.202)	− 0.005 (0.203)	−0.017 (0.196)	− 0.019 (0.155)	0.044 (0.128)
Mixed	−0.054 (0.094)	−0.022 (0.094)	− 0.017 (0.094)	0.004 (0.090)	0.026 (0.071)	0.012 (0.059)
Marital Status (reference: Married/Consensual Union)						
Divorced/Separated	0.257 (0.159)	0.231 (0.160)	0.227 (0.162)	0.243 (0.156)	0.163 (0.124)	0.179 (0.104)
Widower	0.155 (0.161)	0.177 (0.161)	0.190 (0.162)	0.169 (0.156)	0.241 (0.123)	0.117 (0.104)
Single	−0.057 (0.122)	−0.035 (0.121)	−0.025 (0.121)	0.028 (0.118)	0.064 (0.093)	0.030 (0.077)
Education (reference: Illiterate)						
Elementary – Incomplete		0.456 (0.193) **	0.429 (0.196) **	0.462 (0.189) **	0.235 (0.149)	0.191 (0.124)
Elementary – Complete		0.444 (0.202) **	0.441 (0.205) **	0.407 (0.198) **	0.222 (0.157)	0.162 (0.130)
High School – Complete		0.503 (0.222) **	0.489 (0.225) **	0.479 (0.219) **	0.272 (0.174)	0.249 (0.144)
Higher Education – Complete		0.705 (0.260) ***	0.700 (0.261) ***	0.799 (0.253) ***	0.514 (0.200) **	0.408 (0.166) **
Alcohol consumption (reference: No)						
Yes			0.031 (0.094)	0.028 (0.092)	0.049 (0.072)	0.004 (0.060)
Ignored			−0.216 (0.209)	−0.208 (0.203)	−0.181 (0.159)	−0.149 (0.133)
Tobacco use history			−0.260 (0.146)	−0.243 (0.141)	− 0.289 (0.111) **	−0.143 (0.095)
With comorbidity				0.085 (0.085)	0.017 (0.068)	−0.037 (0.056)
Performance Status (reference: PS 0)						
*PS* 1				−0.064 (0.129)	−0.065 (0.101)	− 0.018 (0.086)
*P*S2				−0.441 (0.139) ***	−0.322 (0.110) ***	− 0.148 (0.096)
Staging IV				−0.183 (0.089) **	−0.111 (0.071)	− 0.009 (0.060)
Hospitalization					0.431 (0.068) ***	0.631 (0.061) ***
Chemotherapy/Radiotherapy					0.923 (0.097) ***	0.734 (0.084) ***
Death period (reference: less than 3 months)						
From 3 to 6 months						0.036 (0.082)
From 6 to 9 months						0.259 (0.095) ***
From 9 to 12 months						0.331 (0.105) ***
From 12 to 18 months						0.362 (0.103) ***
Over 18 months						0.951 (0.102) ***
Constant	5.866 (0.287) ***	5.280 (0.366) ***	5.517 (0.388) ***	5.705 (0.401) ***	4.574 (0.329) ***	4.067 (0.279) ***
Adjusted R^2^	0.016	0.032	0.038	0.111	0.449	0.622

Source: Authors’ own work

**p-value < 0.05 ***p-value < 0.01

Standard deviation between brackets

In models 4 and 5, variables related to clinical conditions of patients and the use of some outpatient and hospital services are included. Individuals with lower performance in their daily activities (PS = 2) have lower treatment costs than those with better clinical conditions. There is a positive and significant relationship between hospitalization and the use of radiotherapy or chemotherapy during treatment and the total cost. Evaluation of models’ adjusted R^2^reveals that variables of use of services explain most of the treatment cost variation, once that there is a significant increase in these statistics between models 4 and 5 (0.111 to 0.449). Model 6 shows the effects described in previous models, through patient death time control. There is a positive relationship between cost and survival time, as patients who die between six and nine months after the screening date show on average a treatment cost30% (1-antilog (0.259)) greater than those who die within the period of three months. This effect increases to 44% and 159% when considering patients who died between 12 and 18 months and after 18 months, respectively. In the final model, clinical variables are not statistically significant, and individuals with higher education have costs 50% greater than illiterate patients. Treatment costs of patients who undergo hospitalization and those who make use of chemotherapy or radiotherapy are 88% and 108% higher, respectively, compared to patients who do not make use of such services, even after patient death time control. According to the R^2^ statistic, the explanatory power of the complete model reaches 62%.

In the final model, comorbidity, demographic (marital status, color / race, gender and age) and lifestyle-related variables were also not significant for the determination of the NSCLC care costs. Possibly, tobacco use history has an impact on the likelihood of being affected by the disease. The inclusion of this variable is justified because it seeks to explore the possible presence of other smoking-related diseases, which would have implications for severity and disease patterns among smokers and nonsmokers. In this sense, we used the variable as proxy for other comorbidities associated with tobacco use, such as bronchitis, emphysema and cancers of the mouth, pharynx, larynx and esophagus, for instance.

However, the statistical insignificance of this variable may reflect the low sample variability, to the extent that about 90% of patients studied claimed to have made use of tobacco throughout their lives. The model is shown to be efficient, since no evidence of multicollinearity or heteroscedasticity residuals is observed. According to White test, we cannot reject the null hypothesis of constant variance (White *p*-value = 0.470).The Variance Inflation Factor was 1.85.

## Discussion

This study examines factors associated with treatment cost of 251 patients diagnosed with advanced NSCLC enrolled at INCA in 2011. While cost survey is limited to the time horizon of the study of 18 months observation, some patients died after this period. For 86% of patients – those who died in less than 18 months (*n* = 213) and those who died in a period exceeding 18 months but had a record of referral to the palliative care unit (*n* = 4) – it is assumed that the obtained cost estimates reflect the full treatment of patients. As for the remaining 14% of the sample, we underscore the possibility of cost underestimation. These gather 11 patients with a mean time to death equal to 26 months, and 23 patients with no death record. Still, we consider that estimates presented here provide a satisfactory picture of disease treatment costs, from the perspective of a SUS provider of reference.

The main methodological differences among studies that focus on the treatment of lung cancer costs are due to aspects of the disease itself, the selection of patients, the perspective and time horizon of analysis and potential clinical predictors selected [16–20]. However, some common results are noted. The analysis of the disease treatment costs in the first year after diagnosis in Iran shows that approximately 38% of the total cost is assigned to radiotherapy and drugs and 22% to hospitalization [16]. In substantive terms, such results do not clash with those found in the United States [17]. The NSCLC treatment cost estimate in the Spanish National Health Service shows that major cost components are chemotherapy and surgery [18]. Results presented in this study reinforce these findings and the importance of radiotherapy, chemotherapy and hospitalization as main components of lung cancer-affected patients’ treatment costs.

Regarding treatment cost determinants, even after patient death time control, the multivariate analysis shows that the effects of variables of outpatient and hospital services use are statistically significant and have the greatest weight in total cost variation. Patients who use chemotherapy or radiotherapy or are hospitalized have higher costs. In the absence of the income variable, socioeconomic status is assessed through patient education. Patients with college degrees have the highest costs, perhaps reflecting higher levels of information and communication capacity and demand of treatment resources. Besides that, perhaps they are also more likely to be able to afford the cost of time off work to attend medical appointments or to have employment that permits time off work for such appointments. According to Grossman’s health capital model, education increases efficiency with which individuals produce investments in health, by increasing the level of information, providing greater recognition of health benefits and increasing the ability to give proper follow-up to drugs and treatment instructions [21]. Similar results are found for Medicare patients when using the income variable. A greater payment capacity enables older Americans to benefit from the use of additional services that require some type of copayment, exerting pressure on total program expenditure [20].

In Brazil, however, care offered by INCA is completely free of charge and does not allow copayment arrangements for the provision of additional services. Following the principle of equity, access to health services should take place according to care needs, regardless of socioeconomic status of individuals [22]. Thus, the public hospital of the Brazilian health system should ensure universal access to the population, without any discrimination of services according to ability to pay or instruction level of patients. Cost inequity may reflect inequalities observed in the access and use of health services, especially those related to preventive care [23–26]. In addition to the free services provided by the SUS, private supplementary health insurance is also part of the Brazilian health system and offers the same set of procedures. This enables system double entry by the most advantaged social groups, in that much of its demand for preventive services is met by private health insurance plans. This institutional design creates unequal access to health services, causing poorer people to come to the system with more serious health conditions [27]. In the case of NSCLC, we saw that this entails lower disease treatment costs, whether due to patient deteriorated health condition, which hinders certain types of interventions, or shorter survival time associated with ill-health. Interestingly, however, it is worth noting that even exerting control through all these factors, education remains significant in explaining costs for the more educated group, suggesting some inequity-producing mechanism in the access to cancer care itself. Thus, results point to the need for strategies at all levels of complexity that reduce barriers to access to health services by the population of lower socioeconomic status.

In this study, the variable “tobacco use history” was not consistently significant in the explanation of the disease treatment costs from hospital’s standpoint. However, 90% of the sample declares having made use of tobacco throughout life, thus influencing the parameter’s estimate. It is known that tobacco use is lung cancer’s main risk factor, accounting for over 70% of global deaths from this type of cancer [28, 29]. In Brazil, the strategic tobacco control measures adopted in almost three decades already translate into significant reduction of tobacco use prevalence. Since 1989, with the National Tobacco Control Program coordinated by the Ministry of Health, several actions have been promoted for this purpose, among which stand out the tobacco use implementation and surveillance in collective places and implementation of advertising in the product packaging with information on health risks. In 2011, advances are achieved with the enactment of Law 12.546, which aligns pricing and taxes policies to public health objectives, changing the taxation system on the sale of cigarettes and establishing a minimum price policy for the product. While in 1989, 33.4% of the population aged 18 years and older were smokers, in 2012, this percentage dropped to 12.1% [30]. Thus, in view of the importance of tobacco use as a risk factor for non-communicable diseases and conditions, policies focused on compliance and increased tax mechanisms for the reduction of tobacco use should continue to be encouraged.

It is important to emphasize that these results are not generalizable to the entire Brazilian or state public health system, due to the great inequalities in the structure of healthcare services observed between the regions of the state of Rio de Janeiro and Brazil overall. According to the Cadastro Nacional de Estabelecimentos de Saúde do Ministério da Saúde (*National Registry of Health Establishments of the Ministry of Health*), in 2015, the Northeast region, with the lowest human development index (HDI) in the country, had 12.5 computerized tomography (CT) scanners per 1 million inhabitants. The South region, with the highest HDI, had 22.9. Despite being one of the states with the highest concentration of highly complex services in the country, the State of Rio de Janeiro also reproduces this inequality among its regions. In 2015, while the metropolitan region had 55.3 CT scanners per 1 million inhabitants, the coastal area of Baixada Litorânea had a number equal to 26. Thus, a representative sample of costs for lung cancer treatment in the state or country should consider in its calculation this regional inequality dynamics regarding the supply structure.

However, it is important to highlight: i) the role of INCA as a national reference center for cancer care, acting as an auxiliary institute of the Ministry of Health in the development and coordination of integrated actions for the prevention and control of the disease in Brazil and; ii) the magnitude of the referred hospital in terms of the number of total visits performed in the state. According to the Cancer Hospital Registry System, INCA was responsible for 57% and 3% of the patients with confirmed diagnosis of cancer in the state of Rio de Janeiro and Brazil, respectively, in 2011, the year of the study. Regarding lung cancer specifically, this percentage reaches 74% and 6%, respectively. Therefore, the hospital seems to play a key role in caring for patients with different types of cancer, and especially with lung cancer, at the state level. Since almost 70% of the population of the state depends exclusively on the public health system and this percentage may be even higher when it comes to more complex services, INCA fulfills an important social function in tertiary care and access to cancer services in Rio de Janeiro.

## Conclusion

NSCLC treatment costs inequity observed at a Brazilian public hospital point to the need for strategies that reduce barriers to access and use of health services by the population with lower socioeconomic conditions.

## Abbreviations

C.V.: Coefficient of variation
CT: Computerized tomography
FBH: Brazilian Federation of Hospitals
HDI: Human Development Index
INCA: José Alencar Gomes da Silva National Cancer Institute
NSCLC: Non-small cell lung cancer
OLS: Ordinary least squares
S.D.: Standard deviation
SUS: Unified Health System
